# Urinary MMP-9/UCr association with albumin concentration and albumin-creatinine-ratio in Mexican patients with type 2 diabetes mellitus

**DOI:** 10.7717/peerj.10474

**Published:** 2020-12-16

**Authors:** Víctor Gildardo Arcos-Sacramento, Clara Luz Sampieri, Víctor Hugo Sandoval-Lozano, Rubén Arturo Orozco-Ortega, Mariel Alejandra Acuña-Hernández, Jaime Morales-Romero, Magda Elena Hernández-Hernández, Arturo Rodríguez-Hernández

**Affiliations:** 1 Instituto de Salud Pública, Universidad Veracruzana, Xalapa, Veracruz, México; 2 Secretaría de Salud del Estado de Veracruz, Xalapa, Veracruz, México; 3 Instituto Mexicano del Seguro Social, Unidad de Medicina Familiar No. 10, Xalapa, Veracruz, México

**Keywords:** Chronic Kidney Disease, Diabetes mellitus, Matrix Metalloproteinases

## Abstract

**Background:**

Chronic kidney disease is one of the most common complications of type 2 diabetes mellitus (T2DM), causing an increased risk of cardiovascular morbidity and mortality. Matrix metalloproteinase (MMP) activity has been proposed as useful biomarker for diabetic renal and vascular complications.

**Methods:**

A cross-sectional study was conducted among T2DM patients who attended a public secondary hospital in Mexico. We performed clinical, biochemical, and microbiological assessments, as well chronic kidney disease diagnosis according to the KDIGO guideline. Urinary MMP-9 was quantified by ELISA and adjusted using urinary creatinine (UCr).

**Results:**

A total of 111 patients were included. Most participants were women (66%). Mean age was 61 ± 10 years and median T2DM duration was estimated at 11 years. Through multivariate analysis, MMP-9/UCr was found to be associated with albumin concentration and albumin to creatinine ratio.

**Discussion:**

Validation of non-invasive biomarkers of chronic kidney disease among T2DM patients is necessary. Here, we demonstrate MMP-9/UCr as a potential biomarker of albumin concentration and albumin to creatinine ratio in Mexican patients with T2DM.

## Introduction

The prevalence in Mexico in 2016 of previously diagnosed type 2 diabetes mellitus (T2DM), and the combined prevalence of overweightness and obesity were 9.4% and 72.5%, respectively (Rojas-Martínez et al., 2018). Chronic kidney disease (CKD) is a common complication of T2DM, which confers an increased risk of cardiovascular morbidity and mortality (Górriz et al., 2019). Although urine albumin concentration is normally used to assess kidney disease development and progression among patients with T2DM, high variability of this marker has been observed, as well as insufficient sensitivity and specificity (Norris et al., 2018).

It has been proposed that a molecule involved in the remodeling process during the early pathology of diabetic kidney disease (DKD) would ideally provide the best marker of disease progression (Altemtam, Nahas & Johnson, 2012). An early event of DKD is excessive extracellular matrix (ECM) accumulation, altering the glomerular and tubular basement membranes and mesangial expansion, possibly causing an increased degree of albuminuria, subsequent proteinuria and fibrosis (Morais et al., 2005; Thrailkill, Bunn & Fowlkes, 2009). Recently, urinary matrix metalloproteinase (MMP) activity has been proposed as a sensitive, non-invasive, and clinically useful biomarker for diabetic renal and vascular complications; however, larger scale studies are necessary in order to confirm their utility (Zheng et al., 2012). Matrix metalloproteinase 9 (MMP-9), also known as gelatinase B, is a multidomain zinc enzyme released mainly by inflammatory cells (Bojic et al., 2015; Zhao et al., 2017). MMP-9 activity is closely regulated on multiple levels, including inhibition by regulatory proteins, such as tissue inhibitors of matrix metalloproteinases (TIMP) (Arpino, Brock & Gill, 2015). In addition to extracellular matrix remodeling, MMP-9 regulates the activity of essential and multiple chemokines, cytokines, receptors, growth factors and cell adhesion molecules to induce tubular endothelial-mesenchymal transition, tubulointerstitial fibrosis and inflammation (Vandooren, Van den Steen & Opdenakker, 2013; Zhao et al., 2013; Zhao et al., 2017).

Liquid chromatography-tandem mass spectrometry analysis has revealed the presence of several collagen peptides in urine from T2DM patients, according to the albumin to creatinine ratio (ACR). Moreover, 48 proteases have potentially been involved in the generation of the most important peptides, including MMP-9 (Brondani et al., 2020). In 24-h urine collection, MMP-8 and MMP-9 activity has been shown to be elevated in accordance with the stage of diabetic nephropathy in T2DM patients, compared to healthy age-matched controls (Van der Zijl et al., 2010). In a previous study, we found an association between renal impairment and the presence of urinary MMP-9 in T2DM (García-Tejeda et al., 2018). In the *Mmp-9* gene knockout diabetic mouse model, nephropathic changes such as hypertrophy, increased albuminuria and thickening of the glomerular basement membrane are significantly attenuated (Li et al., 2014). Here, we investigated the possible association between urinary MMP-9 adjusted by urinary creatinine (UCr) and albumin concentration and ACR in Mexican T2DM patients with a diagnosis of CKD.

## Methods

### Design and study population

A cross-sectional study was conducted (November 2016- March 2017) among T2DM patients that were treated in the public second-level unit “Clínica Hospital del Instituto de Seguridad y Servicios Sociales de los Trabajadores del Estado 25 A 300 400” in Xalapa, Veracruz, Mexico. Criteria for inclusion in the study: ≥18 years of age and meet the clinical requirements to estimate the glomerular filtration rate glomerular filtration rate (GFR), according to the KDIGO guideline (Kidney Disease Improving Global Outcomes (KDIGO), CKD Work Group, 2012). Conditions that could affect pre-analytical requirements, as well as levels of MMP-9, were controlled, and subjects presenting menstruation, hematuria, presence of sperm in urine (Hirsch et al., 1992), antibiotic treatment, anuria, liver cirrhosis, rheumatoid arthritis, chronic obstructive pulmonary disease, autoimmune disorder and cancer were excluded, as well as those who had had intercourse or exercised vigorously in the 72 h preceding the clinical sampling. The GFR and ACR were each estimated twice, presenting with the second estimation in each case performed some 3 months after the first. Subjects who did not coincide in terms of GFR and ACR categories were excluded.

### Sampling of subjects

To randomly select subjects, sampling was conducted as previously described in (Hernández-Hernández et al., 2017; García-Tejeda et al., 2018). A convenient size sample was defined as at least 100 participants. To achieve this sample size, a sample frame was defined as at least 290 participants, considering the response and inclusion rates seen in previous studies (Hernández-Hernández et al., 2017; García-Tejeda et al., 2018).

### Ethics

The research protocol was approved by the investigation committee of the “Clínica Hospital del Instituto de Seguridad y Servicios Sociales de los Trabajadores del Estado 25 A 300 400” (Register No. 001/073/0230). This study was conducted according to the principles of the Declaration of Helsinki and included subjects signed an informed consent.

### Clinical, biochemical, and microbiological assessments

Blood samples collection, urine specimens collection, analysis of aerobic bacterial cultures using Cystine-Lactose-Electrolyte Deficient (CLED), anthropometric and vital signs were recorded as previously described in (Hernández-Hernández et al., 2017; García-Tejeda et al., 2018). Glucose, triglycerides, cholesterol, urea and ureic nitrogen were determined in the serum (Cobas C311 Roche). Urine creatinine, urine albumin and seric creatinine were determined using the Jaffé alkaline picrate method (Cobas C311 Roche). Analysis of morning midstream urine specimens were using Combur 10 Test M reagent strips (Cobas U411 Roche). A microscopic examination of urinary sediment was done using Sternheimer-Malbin stain, where 15 fields were analyzed at 40X.

### MMP-9 quantification

Aliquots of urine samples were centrifuged and stored as previously described in (Hernández-Hernandez et al., 2017; García-Tejeda et al., 2018). Human active MMP-9 (82 kDa) and proactive MMP-9 (92 kDa) were measured in urine using a commercially available enzyme-linked immunosorbent assay kit (DMP900 Quantikine; R&D systems, Minneapolis, MN, USA). Each sample, standard curve and controls were assayed following the instructions of the supplier. Blind analysis of the samples was conducted. According to the supplier, the ELISA kit we employed has a sensitivity of 0.156 ng/mL. Intra and inter-assay coefficients of variation were previously established at 5.9% and 7.9%, respectively (García-Tejeda et al., 2018). MMP-9 levels were adjusted by UCr.

### Variables

The T2DM was defined as previous medical diagnosis, which was verified in the medical records. The GFR was estimated using sex-specific CKD Epi equations based on serum creatinine (Kidney Disease Improving Global Outcomes (KDIGO), CKD Work Group, 2012). Urine ACR categories were according to the KDIGO guideline (Kidney Disease Improving Global Outcomes (KDIGO), CKD Work Group, 2012). Subjects were classified as “No CKD” or “With CKD”. No CKD: GFR ≥60 mL/min/1.73 m^2^ (GFR categories G1 and G2) and ACR A1 (normal to mildly increased) present for >3 months, while “With CKD”: GFR ≤59 mL/min/1.73 m^2^ and ACR A1, A2 (moderately increased) or A3 (severely increased) or with GFR ≥60 mL/min/1.73 m^2^ and ACR A2 or A3 present for >3 months. Urinary tract infection (UTI) for women and men were according the SIGN guideline (Scottish Intercollegiate Guidelines Network, 2013). Vigorous exercise was defined as practicing athletics, tennis, swimming, or soccer.

### Statistical analysis

Categorical variables were summarized using absolute frequencies and percentages. Continuous variables of non-normal distribution were expressed through medians and the interquartile range. Correlation analysis was conducted using the Spearman’s rank correlation. Multivariate analysis was performed in order to identify the association between the independent variable, MMP-9/UCr and the dependent variables, albumin concentration and ACR. Three models were generated for each dependent variable. Regression coefficient and CI95% were calculated by multivariate linear regression. A value of *p* ≤ 0.05 was considered statistically significant. EpiDat version 3.1 and IBM-SPSS Statistics, version 23 were used for analysis.

## Results

A total of 292 patients were invited to participate in the study, with signed informed consent obtained from 99% of those invited. Fifty-eight participants were excluded for not meeting the requirements for calculating GFR, antibiotic treatment, anuria, liver cirrhosis, rheumatoid arthritis, or cancer. Subsequently, 75 participants were excluded for having practiced vigorous exercise in the 72 h preceding the clinical sampling (*n* = 1), failure to provide clinical samples (*n* = 14), hematuria (*n* = 58) and sperm in the urine sample (*n* = 2). GFR and ACR were estimated in 158 participants and, more than 3 months later, 137 patients returned for a second estimation of GFR and ACR. The study finally comprised a total of 111 patients with GFR and ACR categories in concordance.

**Table 1:** Comparison of patients with and without chronic kidney disease (CKD).

	**All patients*n* = 111**	**With CKD*n* = 20**	**Without CKD*n* = 91**	***P*-value**
Sex (males), *n* (%)	38 (34.2)	6 (30.0)	32 (35.2)	0.66
Age (years), mean ± SD	61.3 ± 10.3	63.5 ± 10.6	60.8 ± 10.2	0.30
Education level ≤ primary, *n* (%)	35 (31.5)	6 (30.0)	29 (31.9)	0.87
Marital status (without partner), *n* (%)	39 (35.1)	8 (40.0)	31 (34.1)	0.62
Current smoking, *n* (%)	5 (4.5)	1 (5.0)	4 (4.4)	0.99
Current alcoholism, *n* (%)	30 (27.0)	4 (20.0)	26 (28.6)	0.43
Diabetes duration (years), median (P_25_, P_75_)	11.0 (6.0, 17.0)	20.5 (14.3, 25.8)	10.0 (6.0, 15.0)	<0.001
Taking oral hypoglycemic agents, *n* (%)	107 (96.4)	18 (90.0)	89 (97.8)	0.15
Taking insulin, *n* (%)	19 (17.1)	6 (30.0)	13 (14.3)	0.11
With hypertension, *n* (%)	62 (55.9)	12 (60.0)	50 (54.9)	0.68
Urinary tract infection, *n* (%)	8 (7.2)	1 (5.0)	7 (7.7)	0.99
Body mass index (kg/m^2^), median (P_25_, P_75_)	27.9 (25.3, 31.3)	25.7 (24.2, 29.1)	28.4 (25.5, 31.5)	0.013
Waist circumference (cm), mean ± SD				
Males^*^	96.8 ± 7.5	94.4 ± 7.8	97.3 ± 7.5	0.40
Females^**^	95.7 ± 9.1	92.0 ± 9.4	96.6 ± 8.9	0.09
SBP (mmHg), median (P_25_, P_75_)	135 (120, 146)	137 (127, 148)	135 (120, 145)	0.51
DBP (mmHg), median (P_25_, P_75_)	76 (70, 80)	73 (69, 80)	77 (70, 80)	0.30
Serum glucose (mg/dL), median (P_25_, P_75_)	155 (118, 211)	150 (118, 234)	162 (118, 205)	0.67
Serum creatinine (mg/dL), median (P_25_, P_75_)	0.69 (0.58, 0.80)	0.68 (0.52, 1.09)	0.69 (0.59, 0.80)	0.86
Urinary creatinine (mg/dL), median (P_25_, P_75_)	57.2 (36.7, 88.3)	51.1 (30.5, 82.7)	60.3 (41.6, 90.0)	0.11
Urinary albumin (mg/dL), median (P_25_, P_75_)	0.30 (0.19, 0.95)	4.8 (3.2, 11.0)	0.26 (0.19, 0.48)	<0.001
Urinary ACR (mg/g), median (P_25_, P_75_)	5.5 (3.6, 11.3)	77.2 (53.4, 270.3)	5.0 (3.1, 7.3)	<0.001
GFR (mL/min/1.73 m^2^), median (P_25_, P_75_)	97 (88, 102)	92 (57, 108)	98 (89, 102)	0.20
Total cholesterol (mg/dL), mean ± SD	189.6 ± 39.8	186.2 ± 54.9	190.4 ± 36.0	0.75
Triglycerides (mg/dL), median (P_25_, P_75_)	162 (126, 220)	231 (128, 273)	159 (126, 187)	0.09
Urea (mg/dL), median (P_25_, P_75_)	28.4 (23.5, 34.4)	31.2 (25.4, 55.5)	27.7 (23.3, 33.3)	0.03
Ureic nitrogen (mg/dL), median (P_25_, P_75_)	13.0 (11.0, 16.0)	14.5 (12.0, 26.0)	13.0 (11.0, 16.0)	0.02
MMP9/urinary creatinine (ng/ug), median (P_25_, P_75_)	0.0 (0.0, 0.00013)	0.0 (0.0, 0.0011)	0.0 (0.0, 0.0)	0.23
CFU/mL^***^, median (P_25_, P_75_)	0 (0, 8000)	50 (0, 9350)	0 (0, 6600)	0.86
GFR and albuminuria category, *n*				
G1 A1	68	NA	68	NA
G1 A2	9	9	NA	NA
G1 A3	2	2	NA	NA
G2 A1	23	NA	23	NA
G2 A2	3	3	NA	NA
G2 A3	1	1	NA	NA
G3a A2	1	1	NA	NA
G3a A3	1	1	NA	NA
G3b A1	1	1	NA	NA
G3b A2	2	2	NA	NA

**Notes.**

*n* Number of subjects presenting the characteristic of interest ACR albumin to creatinine ratio GFR glomerular filtration rate CFU colony forming units SBP systolic blood pressure DBP diastolic blood pressure NA Not applicable P_25_ 25th percentile P_75_ 75th percentile

Proportions were compared using the chi-square test or Fisher’s exact test. Means were compared by Student’s *t* test. Comparison of medians using the Mann–Whitney *U* test.

* Missing values in “males without CKD” = 1.

** Missing values in “females without CKD” = 7.

*** CLED agar.

### Characteristics of the population

Most of the participants were women and their mean age was 61.3  ± 10.3 years, with 31.5% reported having an education level higher than primary (Table 1). The mean duration of T2DM was 11 years. Most of the patients were prescribed oral hypoglycemic and more than half reported having a diagnosis of hypertension. A total of 18% of the participants had been diagnosed with CKD (Table 1). Markers of kidney damage in urine sediment and granular cylinders were observed in one subject with ACR A2. In the rest of the subjects, no markers of kidney damage were observed. Fasting serum glucose levels were above the expected value for patients with T2DM (70–130 mg/dL) (Table 1). The median value of triglycerides was also above reference values (Table 1).

### Univariate and multivariate analysis

Urinary MMP-9 levels were determinate by ELISA, which were adjusted to UCr. Seventy-four percent of samples had not detectable MMP-9 (Table 1). There was a positive correlation, with a correlation coefficient of at least 0.3230 between the MMP-9/UCr value with age, serum glucose, ACR and CFU (Table 2).

**Table 2:** Correlation between MMP-9/UCr (ng/ug) and general characteristics of participants.

Characteristic	Correlation coefficient	*P*-value
Age (years)	0.2340	0.0135
Type 2 diabetes mellitus (years)	0.1160	0.2240
Body mass index (kg/m^2^)	0.0396	0.6800
Waist size (cm)^*^	0.1190	0.2180
Systolic blood pressure (mm Hg)^**^	0.0052	0.9570
Diastolic blood pressure (mm Hg)^**^	−0.0343	0.7290
Serum glucose (mg/dL)	0.2270	0.0167
Serum creatinine (mg/dL)	−0.0930	0.3310
Urinary creatinine (mg/dL)	−0.1520	0.1110
Urinary albumin (mg/dL)	0.1750	0.0660
Urinary albumin to creatinine ratio (mg/g)	0.2300	0.0154
Glomerular filtration rate (mL/min/1.73m^2^)	−0.1720	0.0702
Total cholesterol (mg/dL)	−0.0198	0.8360
Triglycerides (mg/dL)	0.0218	0.8200
Urea (mg/dL)	0.1800	0.0592
Ureic nitrogen (mg/dL)	0.1780	0.0614
CFU/mL^***^	0.3230	<0.0001

**Notes.**

Correlation analysis was performed using the Spearman method.

CFU colony forming units MMP matrix metalloproteinase UCr urinary creatinine

* Determined in 71 females and in 37 males.

** Determined in 67 females and in 37 males.

*** CLED agar.

Six multivariate models were generated to identify the possible association between urinary MMP-9/UCr and albumin concentration and ACR. Age, body mass index (BMI), waist size, systolic blood pressure, diastolic blood pressure and serum glucose were included in the models (Tables 3 and 4). In unadjusted (*n* = 101), adjusted (*n* = 101), and adjusted in participants without UTI (*n* = 94) models, urinary MMP-9/UCr was the only statistically significant variable in albumin concentration and ACR. BMI also showed a statistically significant association in most of the models. The remaining variables were not found to be significantly associated (Tables 3 and 4).

**Table 3:** Factors associated with albuminuria in participants with type 2 diabetes mellitus.

	Regression coefficient				Partial correlation
	B	CI 95%	Beta	*P*-value	
**Model 1 (not adjusted:n=101)**					
MMP-9/Urinary creatinine (ng/ug)	165.08	19.18, 311.98	0.23	0.0270	0.2280
Urinary creatinine (mg/dL)	−0.001	−0.02, 0.02	−0.01	0.9220	−0.0100
Age (years)	−0.04	−0.16, 0.08	−0.08	0.4920	−0.0720
Body mass index (kg/m^2^)	−0.31	−0.73, −0.12	−0.22	0.1550	−0.1480
Waist size (cm)	−0.01	−0.19, 0.17	−0.01	0.9460	−0.0070
Systolic blood pressure (mm Hg)	0.04	−0.02, 0.11	0.15	0.1830	0.1390
Diastolic blood pressure (mm Hg)	−0.03	−0.16, 0.09	−0.06	0.6080	−0.0540
Serum glucose (mg/dL)	−0.01	−0.02, 0.01	−0.06	0.5650	−0.0600
**Model 2 (adjusted: n=101)**					
MMP-9/ Urinary creatinine (ng/ug)	160.46	24.73, 296.20	0.23	0.0210	0.2310
Body mass index (kg/m^2^)	−0.31	−0.58, −0.05	−0.22	0.0220	−0.2290
**Model 3 (adjusted in participants without UTI: n=94)**					
MMP-9/Urinary creatinine (ng/ug)	383.25	146.30, 620.19	0.31	0.0020	0.3190
Body mass index (kg/m^2^)	−0.30	−0.58, −0.02	−0.21	0.0330	−0.2210

**Notes.**

Dependent variable: urinary albumin (mg/dL).

B, non-standardized coefficient. CI 95%, 95% confidence interval for B. Beta, standardized coefficient. MMP, matrix metalloproteinases. UTI, urinary tract infection.

Model 1: corrected square *R*: 0.04; *P*-value = 0.16; inclusion method of the variables: Enter. Ten participants were excluded for lack of blood pressure or waist size data.

Model 2: corrected square *R*: 0.08; *P*-value = 0.007; inclusion method of the variables: Forward. Model adjusted for the following excluded variables (if *P*-value > 0.05); urinary creatinine (mg/dL), age (years), waist size (cm), systolic blood pressure (mm Hg), diastolic blood pressure (mm Hg) and serum glucose (mg/dL). Ten participants were excluded for lack of blood pressure or waist size data.

Model 3: corrected square *R*: 0.13; *P*-value = 0.001; inclusion method of the variables: Forward. Model adjusted for the following excluded variables (if *P*-value > 0.05): urinary creatinine (mg/dL), age (years), waist size (cm), systolic blood pressure (mm Hg), diastolic blood pressure (mm Hg) and serum glucose (mg/dL).

**Table 4:** Factors associated with urinary albumin to creatinine ratio in participants with type 2 diabetes mellitus.

	Regression coefficient				Partial correlation
	B	CI 95%	Beta	*P*-value	
**Model 1 (not adjusted: n=101)**					
MMP-9/Urinary creatinine (ng/ug)	4319.96	404.66, 8235.26	0.22	0.0310	0.2230
Urinary creatinine (mg/dL)	−0.37	−0.94, 0.20	−0.13	0.1980	−0.1340
Age (years)	−0.83	−3.96, 2.25	−0.06	0.5920	−0.0560
Body mass index (kg/m^2^)	−13.61	−25.01, −2.20	−0.36	0.0200	−0.2400
Waist size (cm)	3.03	−1.80, 7.86	0.19	0.2150	0.1290
Systolic blood pressure (mm Hg)	0.46	−1.29, 2.21	0.06	0.6050	0.0540
Diastolic blood pressure (mm Hg)	−0.06	−3.42, 3.29	−0.005	0.9700	−0.0040
Serum glucose (mg/dL)	−0.17	−0.59, 0.25	−0.09	0.4220	−0.0840
**Model 2 (adjusted: n=101)**					
MMP-9/ Urinary creatinine (ng/ug)	4476.39	798.80, 8153.99	0.23	0.0180	0.2370
Body mass index (kg/m^2^)	−8.45	−15.62, −1.29	−0.23	0.0210	−0.2300
**Model 3 (adjusted in participants without UTI: n=94)**					
MMP-9/Urinary creatinine (ng/ug)	8810.32	2373.36, 15247.27	0.27	0.0080	0.2740
Body mass index (kg/m^2^)	−8.57	−16.07, −1.06	−0.22	0.0260	−0.2310

**Notes.**

Dependent variable: urinary albumin to creatinine ratio (mg/g).

B, non-standardized coefficient. CI 95%, 95 % confidence interval for B. Beta, standardized coefficient. MMP, matrix metalloproteinases. UTI, urinary tract infection.

Model 1: corrected square *R*: 0.06; *P*-value = 0.08; inclusion method of the variables: Enter. Ten participants were excluded for lack of blood pressure or waist size data.

Model 2: corrected square *R*: 0.08; *P*-value = 0.006; inclusion method of the variables: Forward. Model adjusted for the following excluded variables (if *P*-value > 0.05); urinary creatinine (mg/dL), age (years), waist size (cm), systolic blood pressure (mm Hg), diastolic blood pressure (mm Hg) and serum glucose (mg/dL). Ten participants were excluded for lack of blood pressure or waist size data.

Model 3: corrected square *R*: 0.11; *P*-value = 0.002; inclusion method of the variables: Forward. Model adjusted for the following excluded variables (if *P*-value > 0.05): urinary creatinine (mg/dL), age (years), waist size (cm), systolic blood pressure (mm Hg), diastolic blood pressure (mm Hg) and serum glucose (mg/dL).

## Discussion

Through two indicators of CKD, our results show that urinary MMP-9/UCr is associated with albuminuria in patients with T2DM, although detection of MMP-9 was in 26% of the subjects, probably due to the sensitivity of the ELISA. A similar association has been reported by van der Zijl and colleagues between MMP-9 activity and incipient diabetic nephropathy (Van der Zijl et al., 2010). Through an MMP activity assay, van der Zijl and colleagues detected urinary MMP-9 activity in 89% and 74% of increased albuminuria and normal albuminuria patients, respectively. Besides the selection of the technique to determine activity instead of levels, the inclusion criteria employed by van der Zijl and colleagues differed from our study in terms of hematuria, UTI, GFR equation and chronicity. Age, serum glucose and CFU were found to be associated with urinary MMP-9/UCr, which is consistent with the literature (Thrailkill et al., 1999; Morais et al., 2005; Kundu et al., 2013; García-Tejeda et al., 2018).

The pathological mechanism of kidney fibrosis involves a series of complex molecular events that produce an excess of ECM deposition. In response to noxious stimuli, renal cells secrete pro-inflammatory and pro-fibrotic molecules that promote the recruitment of inflammatory cells. This inflammatory state can lead to the epithelial-mesenchymal transition (EMT), thus contributing to the progression of fibrosis (Garcia-Fernandez et al., 2020). Studies have shown that MMP upregulation promotes fibrosis, possibly by interaction with overexpressed TIMPs or as a result of the capacity of degradation of ECM by MMP, the degradative products of which can induce EMT (Garcia-Fernandez et al., 2020). The ECM is a versatile and dynamic compartment that harbors cryptic biological functions that can be activated by proteolysis (Visse & Nagase, 2003), as well as being involved in modulating cell proliferation, migration, differentiation and apoptosis (Sato & Yanagita, 2017).

Type IV collagen is the principal component of the glomerular basement membrane and mesangial matrix. Excretion of type IV collagen in urinary samples has been postulated as an indication of matrix turnover in diseased kidneys (Tomino et al., 2001). Since MMP-9 is involved in the degradation of type IV collagen, changes in its expression or activity might reflect early renal damage in T2DM, prior to any increased level of albuminuria (Van der Zijl et al., 2010). MMP-9 has been associated with vascular kidney damage by diverse pathological mechanisms, including matrix deposition, kidney fibrosis, EMT, arterial stiffening, vascular calcification, and atherogenesis (Provenzano et al., 2020). MMP-9 has been proposed as a potential therapeutic target to prevent kidney fibrosis in CKD (Zhao et al., 2013).

The EMT is characterized by the loss of epithelial markers such as cytokeratin and E-cadherin, and by nuclear translocation of *β*-catenin accompanied by expression of mesenchymal markers such as vimentin and *α*-smooth muscle actin (Tanabe et al., 2020). In patients with T2DM, elevated plasma levels of TGF- *β*1, CXCL-16 and angiopoietin-2 have been shown as independent predictors of albuminuria and these molecular markers can improve renal risk models beyond established clinical risk factors (Scurt et al., 2019). Through the direct inhibition of Mmp-9 using a neutralizing antibody in a murine model of unilateral ureteral obstruction, Tan et al. (2013) have suggested a potential mechanism underlying the contribution of Mmp-9 to kidney fibrosis: (1) Mmp-9 cleaves osteopontin; (2) osteopontin plays a key role in macrophage recruitment; (3) infiltration of macrophages; (4) tubular cell EMT and (5) kidney fibrosis. Moreover, *Mmp-9* can be upregulated by TGF- *β*1 in mouse renal peritubular endothelial cells, causing endothelial–mesenchymal transition (Zhao et al., 2017).

No association was found between urinary MMP-9/UCr and BMI. Among reproductive-aged women, a positive correlation has been demonstrated between some serum MMPs, including MMP-9, and BMI, as well as significantly higher concentrations of MMPs being found in obese subjects, compared to patients of normal BMI (Grzechocińska et al., 2019). Plasma gelatinase MMP-9 activity has been reported as being significantly higher in obese compared to non-obese patients, which could be reversed by bariatric surgery (García-Prieto et al., 2019). A novel function for MMPs as modulators of adipogenesis has been reported as a consequence of abnormal ECM metabolism (Derosa et al., 2008). In animal models, lipid deposition is found in tubular and glomerular portions of the kidneys, the major sites of diabetic nephropathy lesions (Thongnak, Pongchaidecha & Lungkaphin, 2020).

Given the pathophysiological processes that encompass DKD, such as hyperfiltration and pro-fibrotic, pro-inflammatory and angiogenic processes, it has been challenged whether albuminuria, or any other single biomarker, is individually capable of accurately forecasting the development and progression of renal damage in T2DM (Norris et al., 2018). Moreover, a consensus classification of type 1 and type 2 diabetic nephropathies, that covers the entire kidney, states that the early event is injury to the tubules and vessels, rather than glomerular lesions (Tervaert et al., 2010). In our population with G1 and G2 GFR categories, the existence of extra glomerular lesions, and even hyperfiltration, is therefore possible.

The negative association found between BMI with albumin concentration and ACR is conflictive. Higher BMI has been positively correlated with higher ACR and an increased risk of major renal events in patients with T2DM (Mohammedi et al., 2018). Although, Raikou & Gavriil (2019) found that obesity is not a significant risk factor for an increased degree of albuminuria in hypertensive patients with a poor estimated GFR rate, when both diabetes mellitus and a low eGFR value act as confounders.

This study presents some limitations related to the sensitivity of the ELISA kit, and the absence of MMP-9 found in urinary samples might be questionable. Another limitation of this study is the inclusion of patients that received renin-angiotensin system drugs, given their effect on the expression of some MMPs (Lods, 2003). The strengths of this study are the well-defined population and robust statistical analysis. We consider this study is particularly relevant, since CKD prevalence and mortality rate have doubled in Mexico over the last 20 years (Dávila-Torres, González-Izquierdo & Barrera-Cruz, 2015; Salinas Martínez et al., 2020). For this reason, larger-scale studies are required in the Mexican population in order to determine the predictive values of urinary MMP-9/UCr, including sensitivity, positive predictive value, negative predictive value, probability ratio for positive test, probability ratio for negative test and area under the ROC curve. Ideally, these studies should include other kidney disease in order to demonstrate specificity.

## Conclusions

Non-invasive biomarkers of urine are required to assess DKD development and progression. The present study demonstrated that MMP-9/UCr correlated positively with urine albumin concentration and ACR in Mexican patients with T2DM. We propose MMP-9/UCr as a possible early biomarker of CKD in T2DM patients.

##  Supplemental Information

10.7717/peerj.10474/supp-1 Data S1 Raw data

## Additional Information and Declarations

### Data Availability

The following information was supplied regarding data availability: The raw data are available in Supplemental File.
